# Lumbar Transforaminal Epidural Steroid Injection in Patients with Lumbar Radicular Pain; Outcome Results of 2-Year Follow-Up

**DOI:** 10.29252/beat-070209

**Published:** 2019-04

**Authors:** Masoud Hashemi, Payman Dadkhah, Mehrdad Taheri, Mahshid Ghasemi, Ali Hosseinpour

**Affiliations:** 1 *Anesthesiology Research Center, Shahid Beheshti University of Medical Sciences, Tehran, Iran*; 2 *Department of Anesthesiology, Taleghani Hospital, Shahid Beheshti University of Medical Sciences, Tehran, Iran*; 3 *Non-communicable disease research center, Fasa University of Medical Sciences*, *Fasa, Iran*

**Keywords:** Radiculopathy, Steroid injection, Epidural steroid injection, Outcome, Pain management

## Abstract

**Objective:**

To evaluate the effectiveness of transforaminal lumbar epidural steroid injections (TFESI) in patients with unilateral radiculopathy due to lumbar intervertebral disc protrusion regarding pain intensity, functional disability, current opioid intake and patients’ satisfaction.

**Methods:**

The study is conducted in a pain management center (Tehran, Iran), during 2018. Inclusion criteria were age ≥18 years, radiculopathy for more than 6 months due to imagine-proved lumbar intervertebral disc protrusion and no response to conservative treatments. Exclusion criteria were spinal canal stenosis, lumbar surgery, and inability to communicate in Persian language. During a phone call interview, cases were instructed to rate their pain intensity according to the visual analogue scale (VAS), functional ability, satisfaction according to the patient satisfaction score (PSQ) and report current opioid use and additional injection and/or surgery.

**Results:**

Forty-three (89.5%) of the 48 subjects were reachable for study with mean age of 59.14 years and 16 subjects were men (37.2 %). Mean VAS after intervention was 4.67 and before the intervention was 6.91 (*p*=0.002). Mean functional disability before intervention was 47.23 and after intervention was 37 (*p*<0.001). Mean patient satisfaction score was 3.07 while 18 cases reported a PSQ level ≥4.  10 cases reported using opioid for analgesia, 23 cases reported receiving additional TFESIs and 11 reported having undergone lumbar surgery.

**Conclusion:**

Lumbar Epidural steroid injection is an effective non-surgical treatment option with regard to pain relief and improvement in functional ability with an average patients’ satisfaction during 2 years follow up although nearly 25% of patients may need additional injections and half of the patients may finally proceed to surgery.

## Introduction

Among the multiple presentations of back pain and lower extremity pain, lumbar radicular pain is a common condition which may be caused by a herniated intervertebral disc exerting pressure on the nerve root, resulting in pain, functional disability, opioid use for pain relief and consuming health resources. Multiple modalities of treatments are exploding in managing chronic back pain and radiculopathy along with increasing prevalence. Most patients will respond to conservative treatment. But after the failure of conservative treatment, either surgical or nonsurgical modalities such as epidural injections are considered in the management of lumbar radiculopathy [1-3].

Epidural corticosteroid injections (ESIs) have been used for decades as a non-surgical therapeutic modality in controlling of spinal pain and radiculopathy syndromes attributed to intervertebral disc pathology [4]. Epidural injections can be administered through a transforaminal, interlaminar or caudal route, transforaminal ESIs are more specific and selected nerves can be targeted. ESI administered through this route could deposit a larger mass of corticosteroid close to the pain generators at the ventral epidural space allowing a greater degree of drug diffusion, so transforaminal ESI may be more efficacious in alleviating patients’ pain and improving functional ability [5]. However, there is a paucity of evidence for this modality of treatment in managing chronic lumbar radiculopathy and prospective studies have reached varying conclusions about the efficacy of transforaminal epidural steroid injections in the management of pain and functional ability score improvement [6].

This study is designed to evaluate the effectiveness of transforaminal lumbar epidural steroid injections in patients with unilateral radiculopathy due to lumbar intervertebral disc protrusion. This report consists of the results of 43 patients with a 2-year follow-up, and is a continuation of a previously published randomized, double-blind clinical trial on the effects of corticosteroid after lumbar TFESIs. Outcome measures included pain Intensity and functional disability, assessed at baseline and at 24 months following the treatment. Patients’ satisfaction from the intervention and current opioid intake are also evaluated. Additional lumbar spine injection and progression to surgery during the past two years were secondary outcome measures. 

## Materials and Methods

 *Study population*

  The present observational/analytical non-randomized prospective study is designed and conducted in an interventional pain management referral center (Tehran, Iran), during 2018 in order to demonstrate the results from a two-year follow-up of a previously performed intervention of lumbar transforaminal steroid injection on patients with unilateral radicular pain due to lumbar intervertebral disc protrusions. Study protocol was approved by local ethics committee of the university and study was performed in accordance with the ethical standards of the 1964 Declaration of Helsinki.  Study population were all patients who had undergone lumbar TFESIs in our medical center during 2016. Inclusion criteria were age> 18 years, history of unilateral radicular pain for more than 6 months, lumbar intervertebral disc protrusion which was proved by diagnostic medical imaging, and no response to conservative treatments. Exclusion criteria were history of spinal canal stenosis, history of any lumbar surgery, and inability to communicate in Persian language. Sampling was done using convenience method and all the participants of the previous study who met the criteria were included in the present study.

 *Study protocol *

 Baseline and demographic data for all patients were recorded in patient’s profile and each participant is called by an independent researcher. If any patient was unreachable after 3 calls in different times of a day and different days of a week, patient was excluded from the study. 

During the phone call interview, Study aim and objective were described to each patient and participants were instructed to respond to the questions or rate each scale independently. Pain intensity was evaluated based on verbal numerical rating scale (NRS). NRS is one of the most commonly used self-report scales for measuring pain, likely due to its ease of use (it requires no specialized equipment) and because its 0 to 10 metric is preferred by health care professionals. Patients typically were asked, “How strong is your pain during the past 14 days, where 0 is no pain and 10 is the strongest or worst pain you can imagine?” Functional ability was evaluated based on Oswestry Disability Index (ODI). ODI is a self-administered questionnaire measuring “back-specific function” on a 10 item scale with six response categories each. Each item scores from 0 to 5, higher scores being worse, which is transformed into a 0–100 scale. The ten items include pain intensity, personal care, lifting, walking, sitting, standing, sleeping, work, social life and traveling. Patients with scores between 0 to 20 have Minimal Disability, between 21 and 40 have Moderate Disability, between 41–60 have Severe Disability, 61 to 80 are crippled and 81 to 100 are bed-bound or exaggerating their symptoms. Patient Satisfaction was evaluated based on Patient Satisfaction Questionnaire (PSQ). PSQ is a treatment-specific instrument for measuring satisfaction with treatments. Patients were asked to choose their overall rating of TFESI among five choices from excellent to poor. Patients were asked about their Opioid consumption for their presenting symptoms during the past 2 weeks. Additional lumbar spine injection and progression to surgery during the past two years were asked and the answers were documented in their profile.

Due to the long duration of follow-up and for reasons such as death, migration, or change in the status of sample cases over time, the presence of cases with no follow up (loss-to-follow-up) is predictable. To minimize this bias, inclusion and exclusion criteria are limited, and therefore, the samples will be completely homogeneous from the pathological point of view. As a result, the sample population will represent the community studied. In order to avoid recall bias, the primary outcome measured concentrated on current condition of the patients (specifically past 2 weeks).  In order to avoid response bias, patients were provided with adequate details and necessary clarifications about the questions and the correct way of responding to the questionnaires.

 *Statistical analysis*

  Date were analyzed using SPSS (version 18, SPSS Inc., Chicago, IL, USA). Continuous variables are presented as mean (± SD) or median (range) according to the normal or not-normal distribution of data. Ordinal data are presented as count (%). An independent-samples t-test (two-tailed) was used to compare the variables at baseline and at 2-year follow-up between patients. An alpha level <0.05 was considered to be statistically significant. 

## Results

During the past recruitment period (March 2018-December 2018), 48 subjects with unilateral radicular pain had undergone lumbar transforaminal epidural steroid injection in our tertiary medical center. Between 2 years after the procedure, 43 (89.5%) of the 48 subjects were reachable for follow up. Baseline and demographic characteristics of those reachable for follow-up were analyzed. Average age of participants was 59.14 years with 16 subjects being male (37.2 %) and 24 cases (55.8%) reporting no comorbidity. Of 19 cases who had reported comorbidity, 9 were known cases of hypertension (HTN), 4 were known cases of diabetes mellitus (DM) and 1 was known case of ischemic heart disease (IHD) while 2 reported being diagnosed with DM and HTN, 1 reported being diagnosed with IHD and HTN and 2 reported being diagnosed with IHD and HTN at the same time. Baseline data are demonstrated in Table 1. 

**Table 1:** Baseline and demographic data of 43 participants included in the current study

**Variable **	**Value **
**Age (years)**	59.14 ± 13.35
**Gender **	
Men (%)	16 (37.2%)
Women (%)	27 (62.8%)
Comorbidity	
Yes (%)	19 (44.2%)
No (%)	24 (55.8%)
**Disc Level **	
L3/L4 (%)	1 (2.3%)
L4/L5 (%)	14 (32.6%)
L5/S1 (%)	12 (27.9%)
L3/L4 + L4/L5 (%)	3 (7.0%)
L4/L5 + L5/S1 (%)	13 (30.2%)
**Protrusion Site **	
Central (%)	1 (2.3%)
Foraminal (%)	3 (7.0%)
Left Paracentral (%)	18 (41.9%)
Right Paracentral (%)	21 (48.8%)
**Radiculopathy **	
Left lower extremity (%)	20 (46.5%)
Right lower extremity (%)	23 (53.5%)
**Paresthesia (%) **	31 (72.1%)
**Positive Lasek Test (%)**	27 (62.8%)
**Foraminal stenosis (%)**	32 (74.4%)

Upon evaluating the entire results, all 43 cases reported a history of current pain since intervention (ranged from 2-8 in scale) with mean VAS of 4.67. Mean VAS was 6.91 (ranged from 5-10) before the intervention. The difference in VAS before and after intervention was statistically significant. Mean functional disability (ODI score) before intervention was 47.23 (ranged from 33-64). After receiving the intervention and during 2 years follow up, mean ODI score was 37.35 (ranged from 15-68). This difference in ODI was before and after intervention statistically significant. Mean patient satisfaction score (PSQ) was 3.07 while 18 cases reported a PSQ level ≥4. Data are stated in Table 2. Of those who reported having current pain (43 cases), 10 cases reported using opioid for analgesia, 23 cases reported receiving additional TFESIs and 11 reported having undergone lumbar surgery (Table 3).

**Table 2:** Comparison of pain and disability before and after TFESI and patients’ satisfaction score

	**Mean before TFESI**	**Mean 2 years after TFESI** d	***p*** **-value**
**VAS** e	6.91 ± 1.13	4.67 ± 1.44	0.002
**ODI** b	47.23 ± 8.53	37.35 ± 11.3	<0.001
**PSQ** c	NA^a^	3.07 ± 1.18	NA

a **NA: **Not Applicable;

b **ODI:** Oswestry Disability Index;

c **PSQ:** Patient Satisfaction Score;

d **TFESI:** Transforaminal Epidural Steroid Injection;

e **VAS:** Visual Analogue Scale

**Table 3:** Frequency of opioid consumption, additional intervention and surgery

	**Frequency (n=43)**	**Percentage**
**Opioid consumption **	10	23.3%
**Additional TFESI** a	23	53.5%
**Surgery**	11	25.6%

a **TFESI:** Transforaminal Epidural Steroid Injection

## Discussion

The 2 year results of the present study evaluating the effectiveness of lumbar transforaminal epidural steroid injections in patients with unilateral radicular pain due to single-level lumbar intervertebral disc protrusion showed significant, clinically applicable results in interventional pain management settings. This study showed significant improvement in pain relief and functional ability of patients receiving transforaminal epidural steroid at the end of 2 years along with an average patient satisfaction rating (PSQ: 3/5).

 Although epidural steroid injections have been used for more than half a century in the management of lumbosacral radicular pain and numerous studies have evaluated the efficacy of caudal or lumbar administration of epidural steroids, but studies that have specifically assessed the follow-up outcomes regarding opioid consumption, need for additional injections and/or surgery  are still scarce [7-9]. Kennedy *et al*. in 2018 aimed to determine the long-term outcomes for a homogenous group of patients with acute unilateral lumbar radicular pain due to single-level herniated nucleus after lumbar epidural steroid injection at ≥5 years. They found that despite a high success rate at 6 months, the majority of subjects experienced a recurrence of symptoms at some time during the subsequent 5 years. Few reported current symptoms, and a small minority required additional injections, surgery, or opioid pain medications. They concluded that Lumbar disc herniation can be effectively treated in the short-term by TFESI or surgery, but long-term recurrence rates are high regardless of treatment received [10].

In a prospective case series that investigated the outcome of patients with lumbar herniated nucleus pulposus and radiculopathy who received fluoroscopic transforaminal epidural steroid injections, Lutz *et al*. showed that Fluoroscopic transforaminal epidural steroid injections are an effective nonsurgical treatment option for patients with radiculopathy in whom more conservative treatments are not effective [11]. Vad *et al*. compared transforaminal epidural steroid injections with saline trigger-point injections used in the treatment of lumbosacral radiculopathy secondary to a herniated nucleus pulposus. After an average follow-up period of 1.4 years, the group receiving transforaminal epidural steroid injections had a success rate of 84%, as compared with 48% for the group receiving trigger-point injections. They concluded that fluoroscopically guided transforaminal injections can serve as an important tool in the nonsurgical management of lumbosacral radiculopathy secondary to a herniated nucleus pulposus [12].

To identify the short- and long-term therapeutic benefit of fluoroscopically guided lumbar transforaminal epidural steroid injections in patients with radicular leg pain from degenerative lumbar stenosis, Botwin *et al*. performed a prospective cohort study. From a total of 34 patients who were followed for 1 year, Seventy-five percent of patients had successful long-term outcome, reporting at least a >50% reduction between pre-injection and post-injection pain scores. Sixty-four percent of patients had improved walking tolerance, and 57% had improved standing tolerance at 12 months. They concluded that transforaminal epidural steroid injections may help reduce unilateral radicular pain and improve standing and walking tolerance in patients with degenerative lumbar spinal stenosis [13].

Previous studies have reported pain reduction and improved activity after LESI. However, the data on opioid consumption after LESI is less clear.  In a pilot study performed by Sehgal *et al*. 20 patients with chronic low back pain were followed for 3 months after lumbar ESI and pain relief, functional ability and opioid use were evaluated. Over a three month period, they realized that pain ratings improved and opioid use decreased initially after lumbar ESI for LBP but this effect tapered over time [14]. In a study by Butterman *et al*. aiming to determine the efficacy of epidural steroid injection in the treatment of patients with lumbar herniated disc who were surgical candidates revealed that Epidural steroid injection was not as effective as discectomy with regard to reducing symptoms and disability associated with a large herniation of the lumbar disc. However they concluded that epidural steroid injection was effective for up to three years by nearly one-half of the patients who had not had improvement with six or more weeks of noninvasive care [15]. In the present study, more than 50% of participants underwent additional injections and 25% proceeded to lumbar surgery which are consistent with previously performed follow ups. 

Friedly *et al*. in 2008 conducted a 2-year study in which 13,741 different patients underwent an ESI for low back pain. Their aim was to evaluate whether the use of epidural steroid injections (ESIs) is associated with decreased subsequent opioid use in patients and to determine whether treatment with multiple injections are associated with decreased opioid use and lumbar surgery after ESIs. They found that Opioid use did not decrease in the 6 months after ESIs. Patients who received multiple injections were more likely to start taking opioids and to undergo lumbar surgery within the 6 months after treatment with ESIs. They came to the conclusion that ESIs are not reducing opioid use in this population [16]. Findings from our study shows that in a 2 years’ period of follow up, 10 patients (23%) report a current opioid consumption, although due to lack of stratification in surgical and non-surgical patients, correlation between these two groups cannot be evaluated.  Due to overuse of opioids in the management of chronic low back pain  (Although opioids have not been proven to be an effective treatment for chronic low back pain and radiculopathy [17],  it can be predicted that a large number of patients with chronic pain may claim inefficacy or even worsening of their symptoms after pain interventions in order to rationalize their claim for receiving opioids. Besides, Use of opioids after epidural steroid injections may be an expected treatment option for patients in whom the procedure actually “fails” to provide adequate analgesia, therefore authors believe that opioid consumption cannot represent an appropriate outcome measure for success rate assessment of this intervention. However further multi-central studies on lumbar epidural steroid injections with clustered samples can be performed to evaluate a possible association between subsequent surgery and opioid consumption.

 We note some limitations to our study. The study was performed in a single pain intervention department and confirming the results by a relatively small number of patients examined in our study will require further prospective multi-central randomized trials with larger and clustered samples for accomplishing significant, clinically applicable results in interventional pain management settings. 

In conclusion, lumbar Epidural steroid injection is an effective non-surgical treatment option with regard to pain relief and improvement in functional ability with an average patients’ satisfaction during 2 years follow up although nearly 25% of patients may need additional injections and half of the patients may finally proceed to surgery.

## Disclosure

 No commercial party having a direct financial interest in the results of the research supporting this article has or will confer a benefit on the authors or on any organization with which the authors are associated.

## Conflict of Interest:

None declared.

## References

[1] Manchikanti L, Kaye AD, Manchikanti K, Boswell M, Pampati V, Hirsch J (2015). Efficacy of epidural injections in the treatment of lumbar central spinal stenosis: a systematic review. Anesth Pain Med.

[2] anchikanti L, Pampati V, Benyamin RM, Boswell MV (2015). Analysis of efficacy differences between caudal and lumbar interlaminar epidural injections in chronic lumbar axial discogenic pain: local anesthetic alone vs local combined with steroids. Int J Med Sci.

[3] Pandey RA (2016). Efficacy of Epidural Steroid Injection in Management of Lumbar Prolapsed Intervertebral Disc: A Comparison of Caudal, Transforaminal and Interlaminar Routes. J Clin Diagn Res.

[4] Pountos I, Panteli M, Walters G, Bush D, Giannoudis PV (2016). Safety of epidural corticosteroid injections. Drugs in R&D.

[5] hai B, Bansal D, Kay JP, Vadaje KS, Wig J (2014). Transforaminal versus parasagittal interlaminar epidural steroid injection in low back pain with radicular pain: a randomized, double-blind, active-control trial. Pain Physician.

[6] Buenaventura RM, Datta S, Abdi S, Smith HS (2009). Systematic review of therapeutic lumbar transforaminal epidural steroid injections. Pain Physician.

[7] Benoist M, Boulu P, Hayem G (2012). Epidural steroid injections in the management of low-back pain with radiculopathy: an update of their efficacy and safety. Eur Spine J.

[8] Sung MS (2006). Epidural steroid injection for lumbosacral radiculopathy. Korean journal of radiology.

[9] Abram SE (1999). Treatment of lumbosacral radiculopathy with epidural steroids. Anesthesiology:The Journal of the American Society of Anesthesiologists.

[10] Kennedy DJ, Zheng PZ, Smuck M, McCormick ZL, Huynh L, Schneider BJ (2018). A minimum of 5-year follow-up after lumbar transforaminal epidural steroid injections in patients with lumbar radicular pain due to intervertebral disc herniation. Spine J.

[11] Lutz GE, Vad VB, Wisneski RJ (1998). Fluoroscopic transforaminal lumbar epidural steroids: an outcome study. Arch Phys Med Rehabil.

[12] Vad VB, Bhat AL, Lutz GE, Cammisa F (2002). Transforaminal epidural steroid injections in lumbosacral radiculopathy: a prospective randomized study. Spine (Phila Pa 1976).

[13] Botwin KP, Gruber RD, Bouchlas CG, Torres-Ramos FM, Sanelli JT, Freeman ED (2002). Fluoroscopically guided lumbar transformational epidural steroid injections in degenerative lumbar stenosis: an outcome study. Am J Phys Med Rehabil.

[14] Sehgal N, Paidin M, Rasmussen D, Tariq A, Hetzel S (2013). Is there a decrease in opioid use after a single epidural steroid injection in LBP: a pilot study. The Journal of Pain.

[15] Buttermann GR (2004). Treatment of lumbar disc herniation: epidural steroid injection compared with discectomy A prospective, randomized study. J Bone Joint Surg Am.

[16] Friedly J, Nishio I, Bishop MJ, Maynard C (2008). The relationship between repeated epidural steroid injections and subsequent opioid use and lumbar surgery. Arch Phys Med Rehabil.

[17] Deshpande A, Furlan AD, Mailis‐Gagnon A, Atlas S, Turk D Opioids for chronic low‐back pain. Cochrane Database of Systematic Reviews.

